# County Racial Composition and Diminished Associations of Education With Mortality Across Causes

**DOI:** 10.21203/rs.3.rs-11542467/v1

**Published:** 2026-10-30

**Authors:** Gandom Assari, Shervin Assari

**Affiliations:** Orange Coast College; Charles R. Drew University of Medicine and Science

**Keywords:** Education, mortality, racial composition, Minorities’ Diminished Returns, county, health inequalities

## Abstract

**Background:**

Educational attainment is associated with lower mortality, but the health advantages associated with education may depend on social context. Minorities’ Diminished Returns theory provides a framework for examining whether educational resources have weaker associations with population health in counties with higher percentages of Black residents.

**Methods:**

We conducted a repeated county ecological study linking CDC WONDER mortality exports for 2018–2024 with American Community Survey five-year estimates. Outcomes were all-cause mortality, suicide (X60–X84), endocrine, nutritional, and metabolic mortality (E00–E88), and neoplasm mortality (C00–D48). Poisson mean models included population offsets, state and year fixed effects, county-clustered uncertainty, five socioeconomic indicators, racial and ethnic composition, and interactions between each socioeconomic indicator and county percent Black. Education was scaled per 10-percentage-point increase in bachelor’s-degree attainment.

**Results:**

Samples included 21,708 county-years in 3,118 counties for all-cause mortality, 6,807 in 1,193 counties for suicide, 14,383 in 2,332 counties for metabolic mortality, and 20,135 in 2,961 counties for neoplasms. Education tended to have weaker inverse associations with mortality at higher county percent Black. Per 10-percentage-point higher percent Black, the ratio of education rate ratios was 1.016 for suicide (95% CI, 1.004–1.028; P=.011), 1.019 for metabolic mortality (95% CI, 1.0003–1.038; P=.047), and 1.011 for neoplasms (95% CI, 1.002–1.020; P=.021). The all-cause interaction was marginal (ratio, 1.008; 95% CI, 0.9997–1.017; P=.058). At 5% versus 25% Black, higher educational attainment was associated with approximately 9.3% versus 6.4% lower suicide mortality, 13.1% versus 9.8% lower metabolic mortality, and 10.0% versus 8.1% lower neoplasm mortality.

**Conclusions:**

Linear interaction models suggested an MDR-like contextual pattern for education across several mortality outcomes, with a marginal association for all-cause mortality. These exploratory ecological findings warrant replication with age-standardized outcomes and direct measures of structural conditions.

## Introduction

Educational attainment is one of the most widely studied social correlates of health and longevity. ^1,2^ Education may provide access to employment, income, information, social networks, and institutions that support health. ^1,2^ Fundamental cause theory emphasizes that these flexible resources can help people avoid illness and obtain effective care even as specific health risks change. ^1,2^ However, access to a resource does not ensure equal opportunities to use it. ^1,2^ The same educational credential may correspond to different employment conditions, wealth, neighborhood environments, and treatment within institutions. ^1,2^

Minorities’ Diminished Returns (MDRs) theory describes weaker associations of socioeconomic resources with favorable health and other outcomes among marginalized populations. ^3,4,5^ The framework directs attention to the conditions under which resources are translated into health. ^3,4,5^ Structural racism may shape these conditions through housing, education, labor markets, health care, and the distribution of public investment. ^3,4,5^ These processes may persist among people with high educational attainment. MDRs should therefore not be interpreted as evidence that education is unimportant or that disadvantaged populations lack the ability to benefit from it. ^3,4,5^

The question of unequal health returns predates the formal MDRs framework. Farmer and Ferraro found that education was associated with smaller improvements in self-rated health among Black than White adults, providing evidence for a diminishing-returns hypothesis. ^6^ Hudson and colleagues found that reported racial discrimination was associated with depression among African American men with greater education and income. ^7^ In The Price of the Ticket, Hudson, Sacks, Irani, and Asher describe vigilance, pressures associated with upward mobility, and perceived health costs among college-educated African Americans. ^8^ Hudson, Neighbors, Geronimus, and Jackson also report that socioeconomic position–depression associations among African Americans differ by indicator and sex. ^9^ Together, these studies suggest that higher socioeconomic position may coexist with exposures that limit its health advantages; they do not imply that education itself is harmful.

Related scholarship emphasizes the social context of educational resources. Zajacova and Lawrence describe education as both a source of opportunity and a process that can reproduce inequality, with health associations embedded in historical and institutional conditions. ^10^ Hayward, Hummer, and Sasson document variation in education–mortality associations across demographic groups and over time. ^11^ Williams, Priest, and Anderson emphasize that socioeconomic indicators may not represent equivalent resources or experiences across racial groups. ^12^ Bailey and colleagues describe how policies and mutually reinforcing institutional systems shape racial health inequities. ^4,13^ These contributions support examining variation in education associations, although they address different populations, outcomes, and levels of analysis.

Research on education and mortality supports examining racial and social context rather than assuming a uniform gradient. ^14,15^ Zajacova and Hummer examined educational differences in adult mortality across race and sex. ^14,15^ Studies of maternal education and infant mortality have also documented racial inequalities among families with substantial educational resources. ^14,15^ Fishman and colleagues showed that higher maternal education does not eliminate Black–White infant mortality differences. These studies concern individual or family education and motivate a related, but distinct, question about county educational context. ^14,15^

Other MDR research extends this argument beyond mortality. Assari reported weaker inverse associations of maternal education with low birth weight among Black than White births. ^16,17,18^ Research on postponing childbearing and on college graduation has described unequal socioeconomic returns across racial groups. ^16,17,18^ These findings suggest that educational resources may operate within broader systems that shape opportunity, material security, and health. They do not establish the mechanism of a county education association, but they provide a conceptual basis for studying whether that association varies with county racial composition. ^16,17,18^

Past research has examined county racial composition in relation to health and mortality, often alongside economic and institutional conditions. McLaughlin and Stokes evaluated whether minority racial concentration modified the association between income inequality and county mortality, demonstrating the importance of considering race and economic context jointly. ^19^ Hamman reported higher early COVID-19 mortality in counties with larger Black population shares and found that differences in living conditions, health, and work characteristics explained part of that disparity. ^20^ Vilda and colleagues examined county structural racial inequities in relation to infant mortality, illustrating why direct measures of structural conditions add information beyond population composition. ^21^ Together, these studies motivate examining how county racial composition relates to the association between educational resources and health, while recognizing that composition itself is not a measure of racism.

County educational attainment may reflect local employment opportunities, institutional capacity, civic resources, and the availability of health information and services. ^22^ Its association with population mortality may also depend on how those resources are distributed. ^22^ A county with a large percentage of college-educated adults may still have substantial inequalities in school quality, neighborhood conditions, health care access, and economic opportunity. County racial composition provides one contextual measure for examining this variation, although it is not itself a direct measure of structural racism.

We examined whether county percent Black moderated the association between bachelor’s-degree attainment and four mortality outcomes: all-cause mortality, suicide, endocrine/nutritional/metabolic mortality, and neoplasm mortality. ^3,22^ We hypothesized that education would tend to have weaker inverse associations with mortality in counties with higher percent Black. We also examined interactions involving poverty, unemployment, insurance, and home value to assess whether the pattern was specific to education or extended across socioeconomic domains.

## Methods

### Study design and sources

This repeated ecological study linked county mortality records for 2018–2024 with American Community Survey (ACS) five-year estimates ending in the same years. County identifiers were matched using five-digit Federal Information Processing Standards codes. We analyzed public aggregate data without individual identifiers. Reporting follows relevant STROBE principles for observational research. Analyses were exploratory and were not preregistered. ^23,24,25 24^

The mortality exports were obtained from CDC WONDER’s single-race underlying- and multiple-cause mortality databases. The all-cause outcome came from an unrestricted multiple-cause database export; the other outcomes used underlying-cause selections. Database labels describe the data system and do not imply that the all-cause count represents multiple deaths per person. Each outcome was retained as a separate death count and population denominator. We did not combine cause-specific counts to reconstruct all-cause mortality. ^23^

### Outcomes

The four outcomes were all-cause deaths; suicide deaths with underlying-cause codes X60–X84; deaths from endocrine, nutritional, and metabolic diseases with codes E00–E88; and neoplasm deaths with codes C00–D48. Suicide was defined using X60–X84 only; Y87.0 was not included. E00–E88 does not include circulatory-system deaths and should not be labeled combined cardiometabolic mortality. C00–D48 includes malignant neoplasms and other neoplasms, rather than malignant cancer alone. Cause-specific results concern these exact code ranges. ^23^

Deaths and population denominators were used to model mortality. Crude rates were expressed per 100,000 population. Rates were not age-standardized. Age-adjusted county rates were not available in the supplied single-race exports. This distinction is especially important when comparing counties with different age distributions. ^23^

#### Education

The primary exposure was the percentage of adults aged 25 years or older with a bachelor’s degree or higher. We scaled attainment per 10-percentage-point increase. ^24^

#### County racial composition

The moderator was the percentage of county residents classified as non-Hispanic Black alone, also scaled per 10 percentage points. These measures describe the county population and do not identify the education or race of individual decedents. Mortality outcomes included all racial and ethnic groups.

Additional composition covariates were the percentage Latino of any race and the percentage of other non-Hispanic residents, including multiracial residents. Non-Hispanic White share was omitted because the four county composition categories sum to 100%. Changes in one share therefore represent a compositional contrast with White share when the remaining shares are held constant. County percent Black is a measure of population composition, not a proxy that directly quantifies racism or segregation.

### Socioeconomic covariates

Models included poverty, unemployment, uninsured share, and median owner-occupied home value alongside educational attainment. Poverty was the percentage below the poverty threshold; unemployment was measured among civilian labor-force participants; uninsured share was measured among civilian noninstitutionalized residents. Poverty and education were scaled per 10 percentage points, unemployment and uninsured share per 5 points, and home value per doubling using a base-2 logarithm. Home values were nominal. ACS estimates summarize the preceding five years, and adjacent releases overlap substantially. ^24^

### Sample construction and missing data

For each outcome, we required observed deaths, a positive population denominator, and complete ACS socioeconomic and composition measures. Counties needed at least two observed outcome years. Samples differed because outcome suppression and data availability varied by cause. The source exports hid zero and suppressed death rows. We therefore left absent county-year outcomes missing and did not interpret them as zero. Analyses used outcome-specific complete cases without imputation. Exact FIPS matching avoided assuming that changed county boundaries were interchangeable; geographic changes, including Connecticut’s county equivalents, could affect inclusion.

#### Statistical analysis

We fitted Poisson mean models with the logarithm of county population as an offset. Models included state and categorical year fixed effects and county-clustered CR1 sandwich uncertainty. The Poisson specification was used as a model for the mean death count; robust uncertainty did not require equality of the conditional mean and variance. Confidence intervals and two-sided P values used a t distribution with the number of county clusters minus one degrees of freedom. Numerical checks verified convergence and full design rank.^26,27^

First, main-association models included all five socioeconomic indicators and the three composition measures. Second, moderation models added interactions between county percent Black and each of the five socioeconomic indicators simultaneously. Interaction products used mean-centered variables. A joint five-degree-of-freedom Wald test assessed whether the socioeconomic interaction terms collectively differed from zero. Education was the primary interaction of interest; other resource interactions provided context.

Education interactions were summarized as ratios of rate ratios (RRs). A ratio above 1 indicates that the education RR shifts toward higher mortality as percent Black increases. When education has an inverse association, this corresponds to a weaker inverse slope. We estimated education RRs at 5% and 25% Black using linear combinations of the main and interaction coefficients, with uncertainty incorporating their covariance. These percentages were illustrative reporting points, not thresholds defining groups. Figure 1 shows the point-specific estimates, and Table 3 reports the formal continuous interaction tests.

All P values were unadjusted. We describe P=.058 as marginal while reporting its confidence interval and recognizing that it does not meet the conventional .05 threshold. No result was treated as evidence of a racial difference merely because one point-specific association was detectable and another was not. Models with state and year effects combine spatial and temporal variation; they are not estimates of within-county change alone. We did not assess out-of-sample prediction or include heat exposures in these models.

#### Descriptive mortality trends

We also plotted annual crude mortality by county educational attainment and racial composition (Fig. 2). ^23,24^ For each outcome, we restricted this descriptive analysis to counties with observed deaths, positive population denominators, and available education and composition measures in all seven years, 2018–2024. Counties were classified using their mean ACS estimates over that period, so education and composition group membership remained fixed. Education groups were < 20%, 20–<30%, and ≥ 30% of adults with a bachelor’s degree or higher. Composition panels included all counties in the outcome-specific descriptive sample, counties with 0–<10% non-Hispanic Black residents, and counties with 20–<30% non-Hispanic Black residents. The composition bands describe counties near the 5% and 25% model reporting points but are different estimands. Annual rates were total deaths divided by total population within each group, multiplied by 100,000. These population-weighted descriptive rates were unadjusted and were not used to test moderation or changes in educational returns over time.

## Results

### Sample characteristics

The all-cause analysis included 21,708 county-years in 3,118 counties. The suicide analysis included 6,807 county-years in 1,193 counties; metabolic mortality included 14,383 county-years in 2,332 counties; and neoplasms included 20,135 county-years in 2,961 counties. All samples covered 2018–2024. The smaller suicide and metabolic samples reflected outcome-specific availability and suppression. Table 1 presents sample size, pooled crude mortality, and county characteristics. These summaries describe included county-years rather than nationally representative estimates.

### Main socioeconomic associations

Higher educational attainment was associated with lower mortality across all four outcomes in models without interactions. Per 10-point higher bachelor’s attainment, the RRs were 0.924 for suicide, 0.908 for all-cause mortality, 0.879 for metabolic mortality, and 0.911 for neoplasms (Table 2). Higher poverty was associated with higher all-cause and metabolic mortality. Higher unemployment was associated with higher suicide and neoplasm mortality. Higher home value was associated with lower suicide, all-cause, and metabolic mortality. Uninsured share did not show a clear main association with any of the four outcomes after mutual adjustment.

### Education interactions with percent Black

Education tended to have weaker inverse associations with mortality at higher county percent Black. For suicide, the ratio of education RRs per 10-point higher percent Black was 1.016 (95% CI, 1.004–1.028; P=.011). The corresponding ratio was 1.019 for metabolic mortality (95% CI, 1.0003–1.038; P=.047) and 1.011 for neoplasms (95% CI, 1.002–1.020; P=.021). The all-cause interaction was marginal, with a ratio of 1.008 (95% CI, 0.9997–1.017; P=.058). The all-cause interval included 1, and the metabolic estimate was close to the conventional significance boundary.

At 5% Black, a 10-point increase in bachelor’s attainment was associated with approximately 9.3% lower suicide mortality (RR, 0.907; 95% CI, 0.885–0.929). At 25% Black, the corresponding association was approximately 6.4% lower mortality (RR, 0.936; 95% CI, 0.914–0.958). For metabolic mortality, the estimates were 13.1% lower at 5% Black and 9.8% lower at 25% Black. For neoplasms, the estimates were 10.0% and 8.1% lower, respectively. The all-cause estimates were 10.1% and 8.6% lower. Education remained inversely associated with mortality at both reporting points; diminished returns here refers to variation in the magnitude of association, not the absence of an education association.

### Other socioeconomic interactions

The joint tests of the five socioeconomic interactions were detectable for suicide (P=.00033), all-cause mortality (P<.001), metabolic mortality (P=.009), and neoplasms (P<.001). Individual resource patterns differed. A 5-point reduction in unemployment had a weaker inverse association with suicide as percent Black increased (interaction ratio, 1.039; P=.015). Lower poverty had a stronger inverse association with metabolic mortality at higher percent Black (ratio, 0.965; P=.021).

Higher home value showed stronger inverse associations at higher percent Black for suicide, all-cause mortality, and neoplasms. Insurance interactions shifted toward higher mortality for all-cause and neoplasm outcomes as percent Black increased. Because reduced uninsured share did not have a clearly protective association at the lower reporting point, these interactions should not automatically be classified as diminished protective returns. Table 3 places the education findings alongside these mixed domain-specific patterns.

#### Mortality trends by education and county composition

Figure 2 shows consistently lower crude mortality in the higher-education groups across all four outcomes and the three composition panels. The balanced descriptive samples included 3,082 counties for all-cause mortality, 692 for suicide, 1,618 for metabolic mortality, and 2,735 for neoplasms. Overall all-cause rates in the < 20% and ≥ 30% education groups were approximately 1,138 and 768 per 100,000 in 2018, rose to 1,447 and 904 in 2021, and declined to 1,218 and 795 in 2024. Metabolic rates generally increased after 2019, while overall neoplasm rates changed more gradually. Suicide trajectories were less uniform. The < 20% education group in the 20–<30% Black composition panel contained only five counties, limiting interpretation of its fluctuations. County counts for every line appear in the figure. These descriptive education contrasts do not directly test whether the education association differs by county racial composition.

## Discussion

### Principal findings

In this ecological study, county education tended to have weaker inverse associations with several mortality outcomes in counties with higher percentages of Black residents. Formal linear interactions were detectable for suicide, metabolic mortality, and neoplasms, while the all-cause interaction was marginal. The estimated differences were modest but consistent in direction for education. At the same time, other socioeconomic indicators did not show a uniform diminished-returns pattern. The findings therefore support considering education separately from broader claims about every socioeconomic resource.

The main associations also place these findings within the broader literature on social conditions and population health. Higher county education was associated with lower mortality across all four outcomes, while poverty and home value showed associations that varied by outcome. Link and Phelan’s fundamental cause framework, and its later development by Phelan, Link, and Tehranifar, suggest that knowledge, money, power, and social connections can support health through changing disease-specific pathways. ^1,2^ Diez Roux’s work emphasizes that area conditions and individual resources should be distinguished when studying health: a county’s educational composition may reflect local opportunities and institutional resources without measuring any resident’s personal education. ^22^ Her work with colleagues also linked neighborhood disadvantage to coronary disease after adjustment for individual socioeconomic indicators. ^28^These perspectives help explain why an inverse county education association is plausible, but they do not establish that raising county attainment would itself reduce mortality or that our models separate contextual influences from population composition.

### Interpretation in relation to MDRs

The education findings are compatible with an MDR-like contextual pattern. Counties with similar levels of educational attainment may differ in the distribution and accessibility of the opportunities associated with education. A highly educated population does not necessarily imply equal access to stable employment, wealth, health care, or safer environments. Unequal institutional treatment and resource allocation may alter the correspondence between aggregate education and health. MDRs theory offers one interpretation of these differences, but the present analysis does not identify those processes directly. ^3,4,5^

The pattern also relates to Farmer and Ferraro’s finding of smaller education-associated improvements in self-rated health among Black adults and to Hudson and colleagues’ evidence that discrimination may accompany socioeconomic advantage among Black men. ^6,7^ The Price of the Ticket adds qualitative accounts of vigilance and pressures accompanying upward mobility among college-educated African Americans. ^8^ Hudson’s study with Neighbors, Geronimus, and Jackson further cautions against assuming a uniform inverse socioeconomic gradient in depression across indicators and sexes. ^9^ These studies offer possible explanations for unequal health returns, but do not directly test the county mortality interaction examined here. Williams and colleagues caution that similar income or education categories can mask differences in wealth, employment conditions, and exposure to discrimination. ^12^ Bailey and colleagues locate these differences within systems of housing, education, employment, and health care shaped by policy. ^4,13^ This literature supports a structural interpretation worth testing; county percent Black alone cannot identify the relevant institutions, exposures, or mechanisms.

These results extend the question from individual educational returns to county educational context. Individual race, family education, and county composition are different levels of measurement. A weaker county education association at higher percent Black cannot be taken as evidence that the individual education association is weaker for Black decedents than White decedents. It also does not mean that Black population concentration causes higher mortality. Rather, the interaction indicates that one contextual association varies across county composition under the fitted model.

### Comparison with prior research

Previous adult mortality research has emphasized that education gradients vary across demographic groups. Zajacova and Hummer’s work illustrates the need to consider race and sex jointly when studying educational inequalities in mortality. ^14^ Hayward, Hummer, and Sasson further show that these associations vary across groups and historical periods, supporting attention to the social conditions under which education corresponds to longevity. ^11^ Zajacova and Lawrence’s contextual approach also suggests that attainment alone cannot represent all aspects of schooling or the institutions surrounding it. ^10^ Our analysis asks a related geographic question and cannot reproduce the individual-level estimands of these studies. The shared implication is that a single education coefficient may conceal differences in the conditions under which educational resources correspond to health.

Research on maternal education provides a second point of comparison. Fishman and colleagues described persistent racial differences in infant mortality across maternal educational levels, and Assari reported diminished inverse associations of maternal education with low birth weight among Black births. These findings concern birth outcomes and family resources, whereas our outcomes span deaths across ages. Agreement in the direction of unequal education associations supports further investigation, not a common established mechanism across these different outcomes. ^15,16^

Studies of postponing childbearing and college graduation have also described unequal socioeconomic returns among Black and White families. They suggest that education and other life-course resources may translate differently into long-term material security. This literature helps explain why educational attainment alone may not capture access to wealth or supportive institutions. It does not show that those pathways account for the county mortality interactions observed here. ^17,18^

#### Interpretation of the mortality trajectories

The trajectories in Fig. 2 provide descriptive context for the regression findings. Higher-education counties generally had lower crude mortality throughout the study period, while the magnitude and timing of change differed by cause. Hayward, Hummer, and Sasson’s work on group and historical variation in education–mortality associations and Zajacova and Lawrence’s contextual approach support studying educational inequalities within changing social conditions. ^10,11^ Persistent education gradients are also consistent with Link and Phelan’s fundamental cause perspective. ^1,2^ However, the crude gaps in Fig. 2 do not consistently show smaller education contrasts in counties with higher Black population shares. They should not be presented as independent confirmation of MDRs. The descriptive figure compares fixed county groups with different age structures, geographic distributions, and other characteristics; the moderation models estimate conditional associations with education as a continuous measure. These analyses can therefore yield different visual and statistical patterns.

Farmer and Ferraro’s diminishing-returns findings, Hudson’s work on discrimination and the costs of upward mobility, and Williams and Bailey’s accounts of unequal resources and institutional conditions offer possible explanations for the adjusted moderation findings. ^4,6,7,8,9,12,13^ Those pathways were not measured in the trend analysis. In particular, the rise in all-cause and metabolic mortality during 2020–2021 should not be attributed to a specific exposure or mechanism from these data. Fixed group membership reduces changes caused by counties switching categories, but changes in resident populations, selection of counties with complete reported data, and unmeasured age differences remain relevant. The trajectories suggest questions for future age-specific and multilevel analyses rather than establishing changes in the returns to education over time.

### Why results may differ by cause

Education may relate to mortality through several pathways, including employment, health information, access to care, smoking, material conditions, and chronic stress. The relevance of each pathway may differ across suicide, metabolic diseases, and neoplasms. For suicide, local economic opportunities and access to mental health care may be important. For metabolic mortality, prevention, continuity of care, and management of long-term disease may be relevant. Neoplasm mortality combines conditions with different risk factors, detection practices, and treatment opportunities. The broad code grouping limits any disease-specific interpretation.

These are possible explanations rather than measured mediators. Mortality also depends on population age structure, migration, and historical exposures. Because rates were not age-adjusted, counties with older populations may have different mortality regardless of educational context. Age composition may also vary with percent Black and educational attainment, producing apparent interactions. The marginal all-cause finding deserves particular caution because all-cause deaths combine outcomes with different age patterns and education gradients.

### Other socioeconomic resources

The contrasting home-value and poverty interactions argue against describing every resource as having diminished returns in counties with higher percent Black. Home values may reflect local housing markets, population composition, and economic conditions rather than accessible household wealth. Insurance coverage measures availability of coverage, not care quality, continuity, or affordability. Mutually adjusted coefficients describe each domain conditional on the others. Correlation among socioeconomic indicators may also change their interpretation. These considerations may help explain why education, employment, poverty, insurance, and housing did not share the same moderation pattern.

### Implications

The findings suggest that policies addressing educational attainment may need to be paired with attention to the institutions through which education can support health. Potential areas include equitable employment opportunities, affordable and high-quality care, fair housing, and public investment in underserved communities. The study does not test the effects of these policies. Their relevance follows from broader evidence on structural racism and health and from the possibility that equal educational attainment does not ensure equal access to health-supporting conditions. ^4,5^

Future work should directly measure institutional access, segregation, resource distribution, and discrimination rather than relying on population composition to characterize context. Linking individual educational and clinical information to county conditions would help distinguish family resources from geographic resources. Age-standardized and age-specific outcomes are essential next steps. Analyses should also assess whether the contextual pattern holds across alternative samples and time periods.

### Limitations

First, the ecological design cannot establish individual educational returns, racial differences among decedents, or causation. No individual education, clinical risk, behavior, treatment, or cause-specific disease history was available. Second, rates were not age-standardized and models did not adjust for county age composition. This is a major limitation for cross-county mortality comparisons and may account for part of the observed associations or moderation.

Third, suppressed and zero rows were hidden in the mortality exports. Complete-case analysis therefore selects counties and years with observable outcomes, particularly for suicide. Missing values were not treated as zero, but exclusion can still produce selection bias. Samples differed across outcomes, so differences in estimated moderation cannot be attributed solely to cause of death.

Fourth, ACS five-year estimates overlap across adjacent releases and include sampling uncertainty that was not propagated into the models. County socioeconomic averages do not measure resource distribution within counties. Nominal home values may reflect price changes rather than changes in accessible wealth. Geographic changes and exact FIPS matching can affect coverage.

Fifth, percent Black is not a direct measure of structural racism, and residual confounding by age, urbanization, regional conditions, institutional resources, migration, and other contextual factors remains possible. State and year effects address broad differences but do not remove all between-county confounding. County-clustered uncertainty accommodates repeated observations within counties, not spatial dependence between counties.

Finally, the analyses and interaction tests were exploratory. All P values were unadjusted, and the marginal all-cause interaction does not meet the conventional .05 threshold. The borderline metabolic result and other nominal associations require replication. The models characterize conditional associations and were not evaluated as predictive tools. Our interpretation is limited to the stated linear interaction specification and should not be read as evidence of a threshold.

## Conclusions

County education tended to have weaker inverse associations with suicide, metabolic, and neoplasm mortality at higher county percent Black, with a marginal pattern for all-cause mortality. These findings are compatible with contextual MDRs but do not establish individual racial differences or causal effects. Unequal access to the opportunities associated with education is a plausible interpretation that requires direct investigation. Replication with age-standardized outcomes and measures of structural conditions is needed before drawing stronger conclusions.

## Figures and Tables

**Figure 1:**
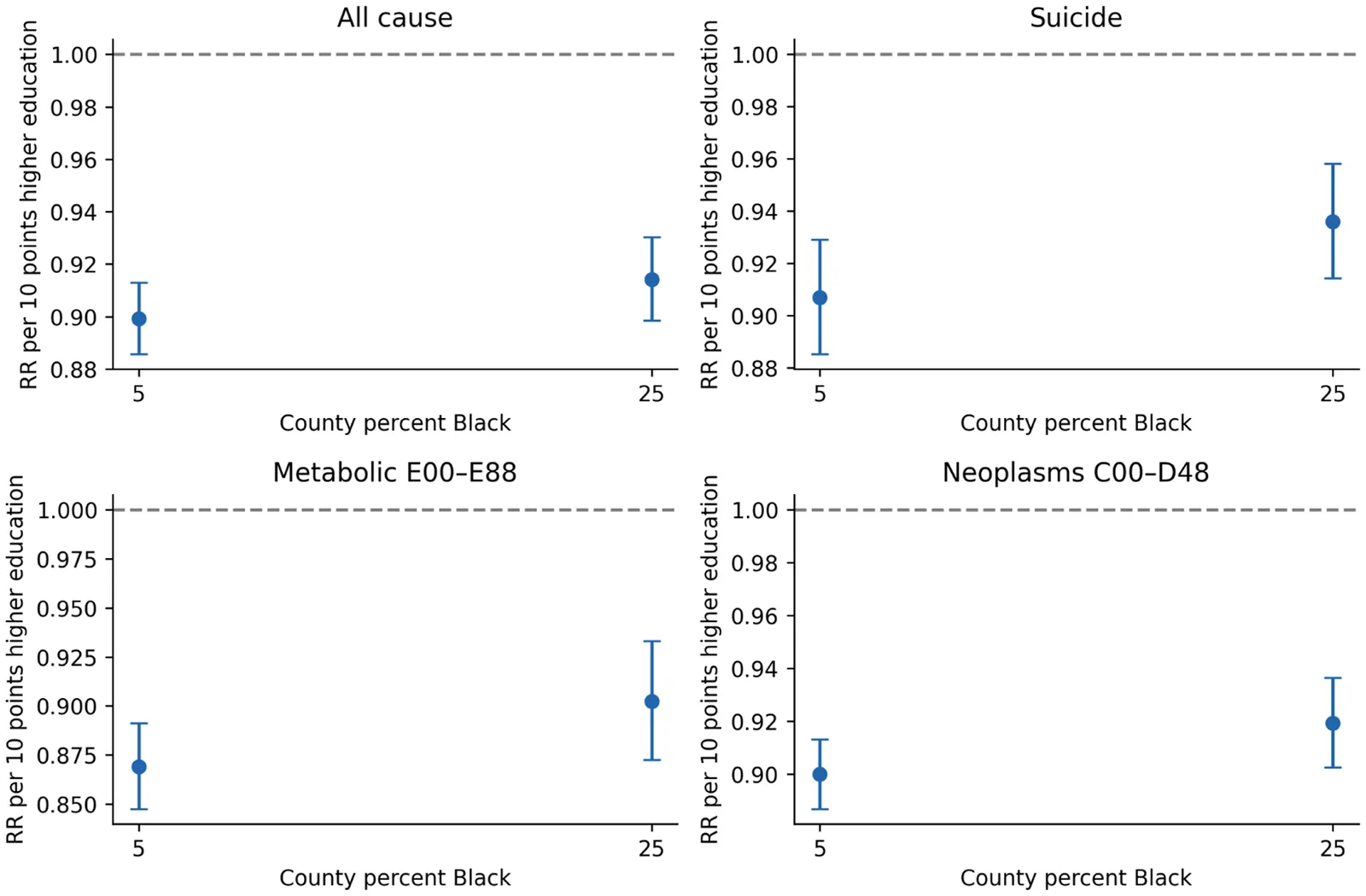
Education Associations at Two County Racial Compositions *Note.* Education rate ratios per 10-point higher bachelor’s-degree attainment at 5% and 25% non-Hispanic Black county population share. Bars show 95% confidence intervals. The dashed line marks RR=1. Predictions derive from the linear interaction models in Table 3. Reporting points are illustrative composition values, not categories or thresholds. Mortality includes residents of all races; these are not race-specific education effects.

**Figure 2:**
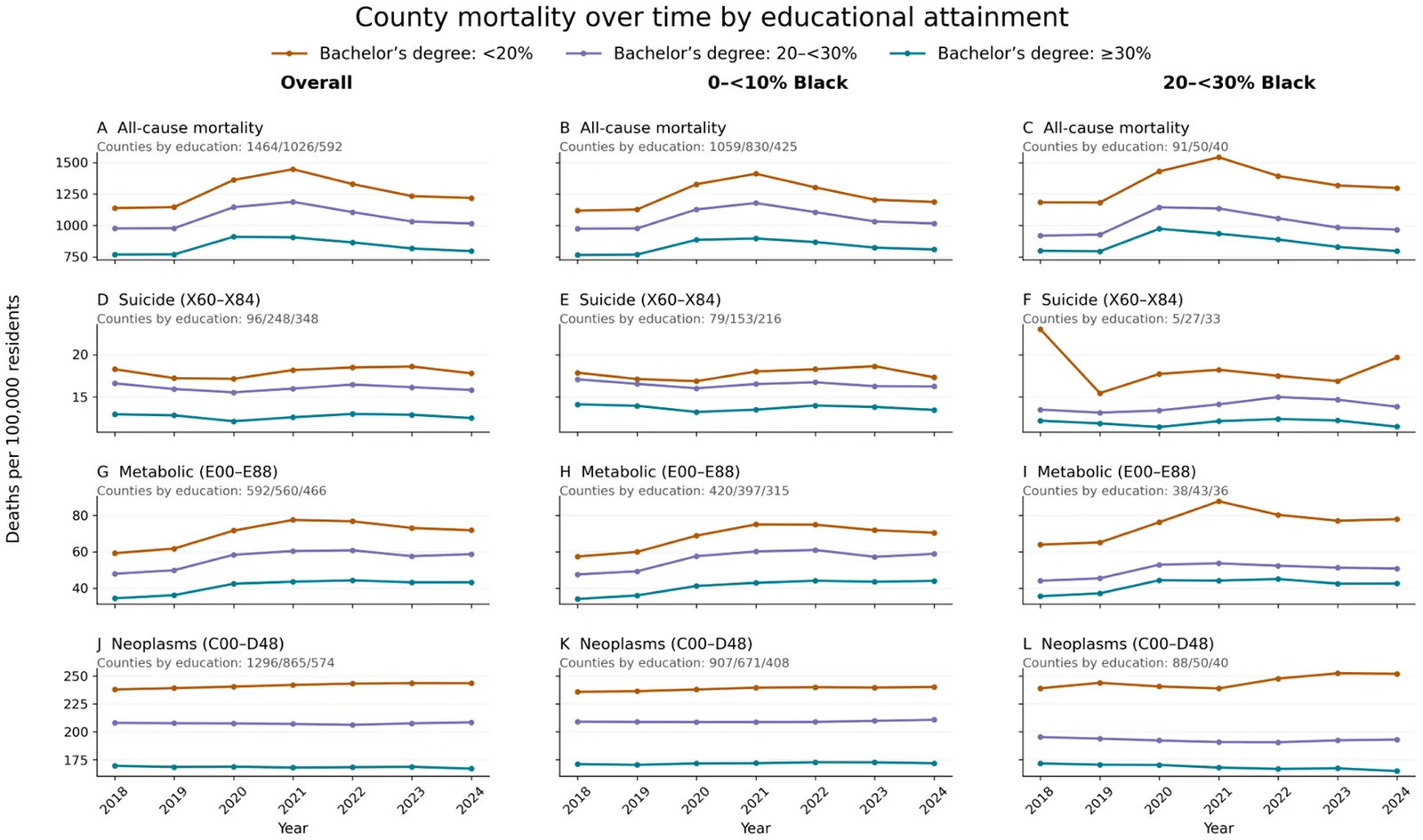
Mortality Trends by Education and County Racial Composition **Note.** Annual observed crude mortality per 100,000 residents, 2018–2024. Rows show all-cause mortality, suicide (X60–X84), metabolic mortality (E00–E88), and neoplasms (C00–D48); columns show the overall descriptive sample, 0–<10% non-Hispanic Black counties, and 20–<30% non-Hispanic Black counties. Lines denote <20%, 20–<30%, and ≥30% bachelor’s-degree attainment. Group membership uses each county’s mean ACS estimates over 2018–2024. Each outcome includes counties with reported data in all seven years. Rates pool deaths and population within each group. County counts follow the legend order. Rates are unadjusted and are not age-standardized. Composition bands are descriptive categories, not the exact model reporting points in Figure 1. The low-education suicide line in the 20–<30% Black panel includes five counties.

**Table 1:** Sample Characteristics

Characteristic	All cause	Suicide	Metabolic E00– E88	Neoplasms C00–D48
County-years	21,708	6,807	14,383	20,135
Counties	3,118	1,193	2,332	2,961
Deaths	21,881,821	284,295	1,084,557	4,330,016
Pooled crude rate per 100,000	945.7	14.1	48.4	187.5
Bachelor attainment (%)	23.0 (9.9)	29.5 (10.8)	24.4 (10.4)	23.2 (10.1)
Non-Hispanic Black (%)	8.9 (14.3)	9.6 (11.7)	10.0 (14.1)	9.4 (14.6)
Poverty (%)	14.7 (6.1)	13.1 (4.7)	14.7 (5.6)	14.8 (6.0)
Unemployed (%)	5.1 (2.6)	5.3 (1.7)	5.4 (2.1)	5.2 (2.3)
Uninsured (%)	9.5 (5.0)	8.6 (4.1)	9.1 (4.5)	9.3 (4.7)
Median home value ($)	180,950.5 (119,204.1)	246,197.6 (147,486.4)	197,515.0 (122,263.1)	184,834.8 (121,212.0)

*Note.* Means (SDs) are unweighted across county-years. Pooled crude rates divide total deaths by summed annual population denominators. Samples cover 2018–2024 and differ by outcome availability. Counts are selected-sample summaries, not national totals. Home values are nominal.

**Table 2:** Main Socioeconomic Associations With Mortality

SES indicator and increment	All cause	Suicide	Metabolic E00– E88	Neoplasms C00– D48
Poverty + 10 points	1.079 (1.043–1.116) P = 1.1e-05	1.053 (0.999–1.109) P = 0.0526	1.135 (1.077–1.197) P = 3.05e-06	1.028 (0.991–1.066) P = 0.146
Bachelor attainment + 10 points	0.908 (0.895–0.922) P = 7.3e-38	0.924 (0.905–0.944) P = 9.58e-13	0.879 (0.860–0.898) P = 4.89e-30	0.911 (0.897–0.926) P = 2.57e-29
Unemployment + 5 points	1.023 (0.993–1.054) P = 0.132	1.098 (1.037–1.162) P = 0.00137	0.957 (0.907–1.010) P = 0.111	1.037 (1.003–1.072) P = 0.0328
Uninsured + 5 points	0.998 (0.975–1.021) P = 0.87	1.018 (0.977–1.060) P = 0.399	0.972 (0.938–1.007) P = 0.12	1.009 (0.985–1.034) P = 0.449
Home value doubled	0.930 (0.899–0.961) P = 1.98e-05	0.932 (0.878–0.989) P = 0.021	0.862 (0.816–0.910) P = 7.8e-08	0.972 (0.930–1.015) P = 0.193

*Note.* Rate ratios (95% CIs) from separate outcome models without interaction terms. All five SES measures and three composition shares are entered together with state and year effects. County-clustered uncertainty; P values unadjusted. These models do not provide age-standardized rates.

**Table 3:** Socioeconomic Interactions With County Percent Black

SES improvement	All cause	Suicide	Metabolic E00–E88	Neoplasms C00– D48
Poverty 10 points lower	0.9963 (0.9840–1.0087) P = 0.555	1.0099 (0.9857–1.0347) P = 0.425	0.9648 (0.9358–0.9947) P = 0.0213	0.9977 (0.9840–1.0116) P = 0.744
Bachelor attainment 10 points higher	1.0083 (0.9997–1.0169) P = 0.0583	1.0159 (1.0036–1.0283) P = 0.0114	1.0189 (1.0003–1.0379) P = 0.0468	1.0107 (1.0016–1.0200) P = 0.0213
Unemployment 5 points lower	1.0046 (0.9944–1.0149) P = 0.379	1.0390 (1.0075–1.0715) P = 0.0148	0.9907 (0.9629–1.0194) P = 0.521	1.0007 (0.9892–1.0124) P = 0.899
Uninsured share 5 points lower	1.0211 (1.0110–1.0314) P = 4.33e-05	1.0078 (0.9911–1.0248) P = 0.361	1.0118 (0.9930–1.0309) P = 0.221	1.0155 (1.0050–1.0261) P = 0.00361
Home value doubled	0.9716 (0.9579–0.9854) P = 6.54e-05	0.9659 (0.9425–0.9898) P = 0.00549	0.9782 (0.9516–1.0057) P = 0.119	0.9623 (0.9462–0.9786) P = 7.61e-06
Joint interaction P	1.81e-08	0.000329	0.00935	1.22e-07

*Note.* Ratios of favorable-SES RRs (95% CIs) per 10-point higher county percent Black. A ratio above 1 means the favorable-SES slope shifts toward higher mortality; this represents diminished protection only when an inverse association is present. Models include all five interactions simultaneously, all main terms, and state/year effects. Joint tests assess the five interaction coefficients. P values are unadjusted; the all-cause education P=.058 is described as marginal and its CI includes 1.

## Data Availability

The linked dataset, numerical estimates, and analysis scripts have been prepared. Original data sources are CDC WONDER and the US Census Bureau.
